# Supplementary material for: A case report of multiple primary prostate tumors with differential drug sensitivity
Source: Nat Commun. 2020 Feb 13;11:837. doi: 10.1038/s41467-020-14657-7 (PMC7018822; doi:10.1038/s41467-020-14657-7)
Supplement: Supplementary file 1 — Supplementary Information [file 41467_2020_14657_MOESM1_ESM.pdf]

A case report of multiple primary prostate tumors with differential drug sensitivity

Wilkinson et al.

Supplementary Information

Supplementary Figures
Supplementary Tables

Supplementary Figure 1

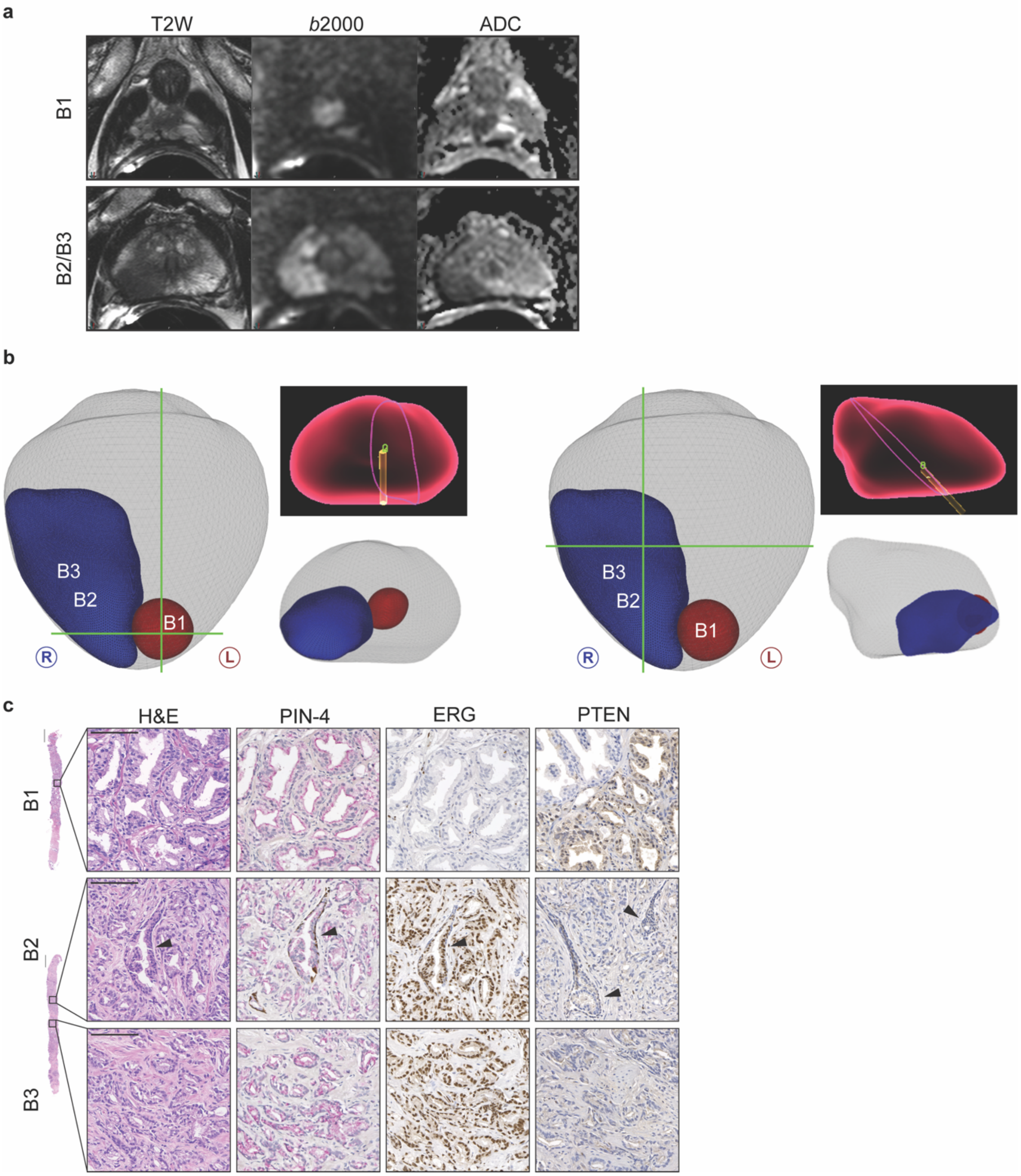

Supplementary Figure 1. Pre-treatment biopsies targeting right- and left-sided prostate cancers. a, Representative slice through left distal apical component (B1) and right apical component (B2/B3, sampled posteriorly on biopsy) as shown on T2-weighted (T2W), high *b*-value (*b*2000), and apparent diffusion coefficient (ADC) MRI. **b,** Targeted biopsies showing the target and UroNav location of the left-sided (B1) Gleason 7 (4+3) biopsy and right-sided (B2/B3) Gleason 9 (4+5) biopsy. **c**, Immunohistochemical staining of left-sided B1 and right-sided B2/B3 biopsies for H&E, PIN-4 cocktail, anti-ERG, and anti-PTEN. Arrowheads indicate IDC-P tumor. Biopsy scale bar: 1000 µm. Inset scale bar: 100 µm.

Supplementary Figure 2

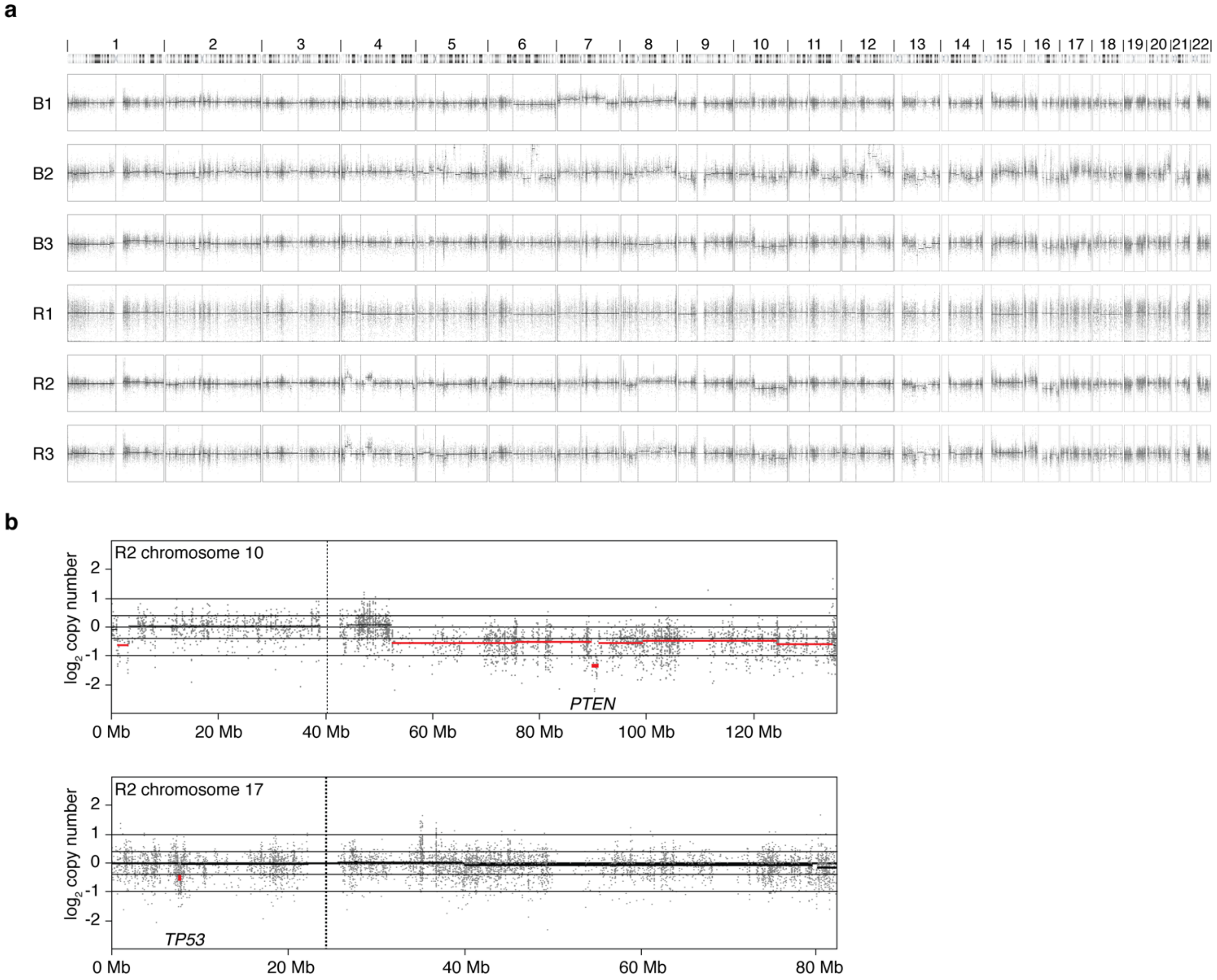

Supplementary Figure 2. Copy number profile of pre- and post-treatment tumor foci. **a,** B1: biopsy from left side. B2: biopsy from right side, IDC-P histology. B3: biopsy from right side, invasive histology. R1: residual tumor from left side. R2: residual tumor from right side, IDC-P histology. R3: residual tumor from right side, invasive histology. Data shown is depicted as $\log_2$ copy number ratio (tumor vs. normal) with the center axis at 0. **b**, Higher resolution whole-chromosome $\log_2$ copy number ratio plots of chromosomes 10 and 17 from the IDC-P focus of residual tumor from the right side, depicting the first and second copy losses of *PTEN* (chromosome 10) and the focal deletion of *TP53* (chromosome 17). Vertical dotted lines depict the location of the centromeres.

Supplementary Figure 3

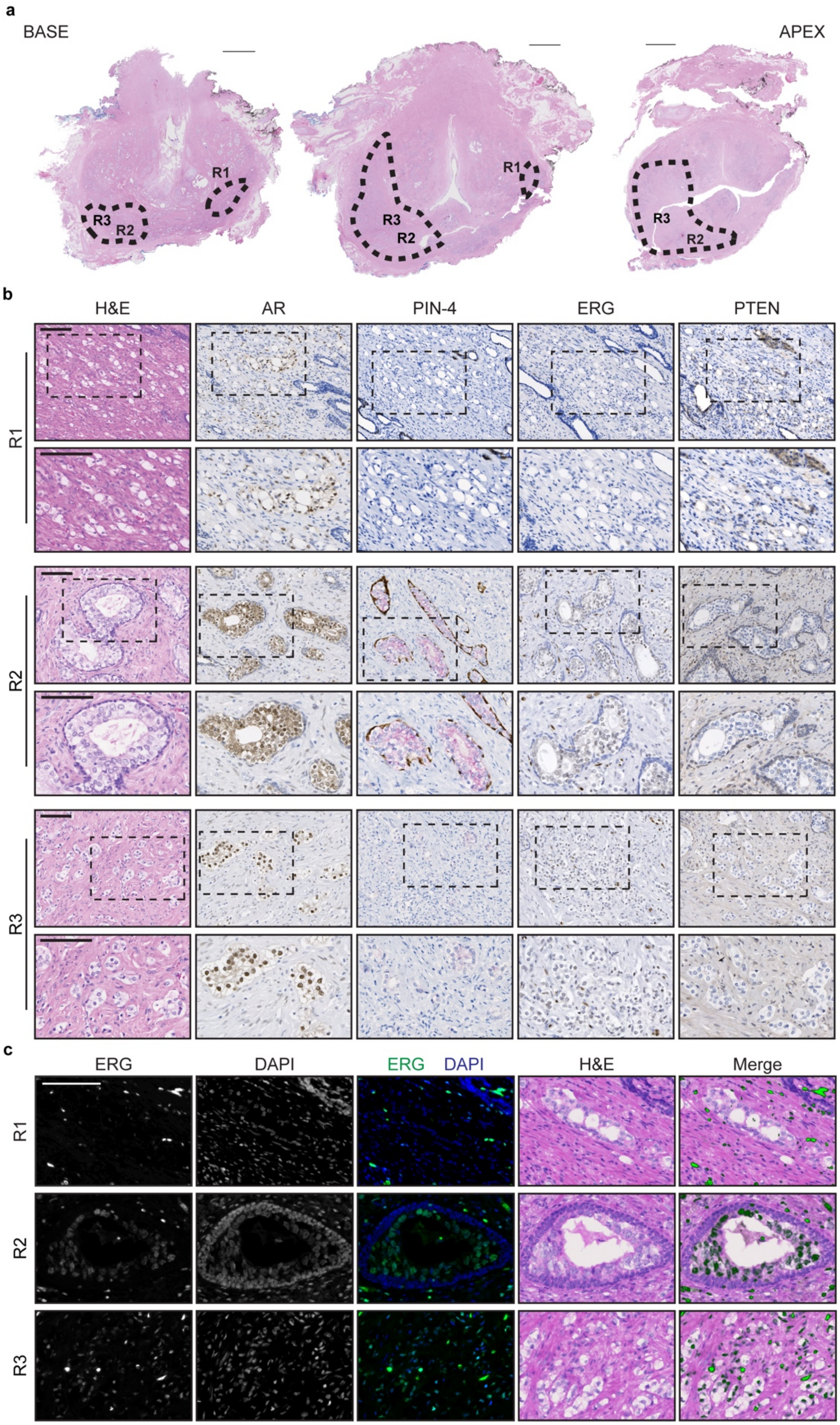

Supplementary Figure 3. Residual tumor histopathologic profile. a, Residual tumor was mapped on whole-mount pathology, showing two regions of tumor foci corresponding to the left- (R1) and right-sided (R2/R3) tumors identified at baseline across three blocks of

tumor used for laser capture microdissection. **b**, Representative histopathological staining of H&E, anti-AR, PIN-4 cocktail, anti-ERG, and anti-PTEN. Insets from boxed regions are shown below each panel. **c**, Opal immunofluorescent staining for anti-ERG, DAPI, and merged anti-ERG/DAPI. After scanning, the slide coverslip was removed, stained for H&E and scanned again, and anti-ERG immunofluorescence was merged onto the H&E image. Scale bars: Whole mount, 5 mm; insets, 100 μm.

Supplementary Table 1. Multiparametric MRI acquisition parameters

Parameter	T2 Weighted	DWI	High *b*-Value DWI	DCE MR Imaging
Field of view (mm)	140 × 140	140 × 140	140 × 140	262 × 262
Acquisition Matrix	304 × 234	112 × 109	76 × 78	188 × 96
Repetition time (msec)	4434	4986	6987	3.7
Echo time (msec)	120	54	52	2.3
Flip angle (degrees)	90	90	90	8.5
Section thickness (mm), no gaps	3	3	3	3
Image reconstruction matrix (pixels)	512 × 512	256 × 256	256 × 256	256 × 256
Reconstruction voxel imaging resolution (mm per pixel)	0.27 × 0.27 × 3.00	0.55 × 0.55 × 2.73	0.55 × 0.55 × 2.73	1.02 × 1.02 × 3.00
Time for acquisition (min:sec)	2:48	4:54	3:50	5:16

Supplementary Table 2. Summary of selected somatic copy number alterations

Focus	PGA	Gene	log_2 CN ratio	Cytoband(s) of alteration(s)
B1	7.39%	*PTEN*	0.01	N/A
		TP53	-0.07	N/A
		BRCA1	-0.06	N/A
		BRCA2	0.01	N/A
		ATM	-0.03	N/A
B2	22.04%	*PTEN*	-0.61	10q21.1-26.3
		TP53	-0.83	17p13.1
		BRCA1	0.15	N/A
		BRCA2	-0.21	N/A
		ATM	-0.64	11q14.3-25
B3	8.64%	*PTEN*	-0.49	10q21.1-26.3
		TP53	-0.49	17p13.1
		BRCA1	-0.68	17q12-21.31
		BRCA2	0.01	N/A
		ATM	0.06	N/A
R1	0.85%	*PTEN*	0.04	N/A
		TP53	-0.06	N/A
		BRCA1	0.01	N/A
		BRCA2	-0.03	N/A
		ATM	0.07	N/A
R2	6.72%	*PTEN* *	-0.53; -1.33	10q21.1-26.3; 10q23.31
		TP53	-0.52	17p13.1
		BRCA1	-0.08	N/A
		BRCA2	-0.08	N/A
		ATM	0.03	N/A
R3	5.81%	*PTEN*	-0.48	10q21.3-26.3
		TP53	-0.61	17p13.1
		BRCA1	-0.02	N/A
		BRCA2	0.01	N/A
		ATM	0.01	N/A

The log_2 copy number ratio for 5 selected loci are shown, along with cytoband(s) containing the resolved deletion that encompasses the gene of interest. PGA: percentage of genome altered. * Two values are shown for *PTEN* in sample R2 representing both chromosomes: the first larger arm-level deletion on one chromosome and a small focal deletion on the other chromosome.

Supplementary Table 3. Summary of point mutation focality from exome sequencing

Focality	Number of point mutations
Only in B1	152
Only in B2	210
Only in B3	66
Shared by B2/B3	9
Shared by B1 and B2 and/or B3	0
Only in R1	1
Only in R2	266
Only in R3	149
Shared by R2/R3	8
Shared by B1/R1	0
Shared by B2/R2	2
Shared by B3/R3	0
Shared by B2/R2/R3	1
Shared by B1/B2/B3/R1/R2/R3	0

Source data are provided in a source data file.

Supplementary Table 4. Summary of point mutation focality from targeted resequencing

Sampling	Number of point mutations
Selected for validation	51
Removed due to MAPQ < 10	5
Failed to amplify	1
Detected mutation in Normal	5
Detected mutation only in B1	0
Detected mutation only in B2	1
Detected mutation only in B3	0
Detected mutation only in B2/B3	0
Detected mutation only in R1	0
Detected mutation only in R2	1
Detected mutation only in R3	0
Detected mutation only in R2/R3	5
Detected mutation only in B1/R1	1
Detected mutation only in B2/R2	1
Detected mutation only in B3/R3	0
Detected mutation only in B2/R2/R3	13
Detected mutation only in B3/R2/R3	3
Detected mutation only in B2/R2/B3/R3	4
Detected mutation in B1/B2/B3/R1/R2/R3	0
Not detected in any sample	11

Source data are provided in a source data file.
