# Supplementary material for: A case report of multiple primary prostate tumors with differential drug sensitivity
Source: Nat Commun. 2020 Feb 13;11:837. doi: 10.1038/s41467-020-14657-7 (PMC7018822; doi:10.1038/s41467-020-14657-7)
Supplement: Supplementary file 3 — Reporting Summary [file 41467_2020_14657_MOESM3_ESM.pdf]

nature research

Corresponding author(s): Adam Sowalsky

Last updated by author(s): 1/15/2020

Reporting Summary

Nature Research wishes to improve the reproducibility of the work that we publish. This form provides structure for consistency and transparency in reporting. For further information on Nature Research policies, see Authors & Referees and the Editorial Policy Checklist .

Statistics

For all statistical analyses, confirm that the following items are present in the figure legend, table legend, main text, or Methods section.

n/a	Confirmed	
☐	☒	The exact sample size (*n*) for each experimental group/condition, given as a discrete number and unit of measurement
☐	☒	A statement on whether measurements were taken from distinct samples or whether the same sample was measured repeatedly
☒	☐	The statistical test(s) used AND whether they are one- or two-sided<br>*Only common tests should be described solely by name; describe more complex techniques in the Methods section.*
☒	☐	A description of all covariates tested
☒	☐	A description of any assumptions or corrections, such as tests of normality and adjustment for multiple comparisons
☒	☐	A full description of the statistical parameters including central tendency (e.g. means) or other basic estimates (e.g. regression coefficient) AND variation (e.g. standard deviation) or associated estimates of uncertainty (e.g. confidence intervals)
☒	☐	For null hypothesis testing, the test statistic (e.g. *F*, *t*, *r*) with confidence intervals, effect sizes, degrees of freedom and *P* value noted<br>*Give P values as exact values whenever suitable.*
☒	☐	For Bayesian analysis, information on the choice of priors and Markov chain Monte Carlo settings
☒	☐	For hierarchical and complex designs, identification of the appropriate level for tests and full reporting of outcomes
☒	☐	Estimates of effect sizes (e.g. Cohen's *d*, Pearson's *r*), indicating how they were calculated

Our web collection on statistics for biologists contains articles on many of the points above.

Software and code

Policy information about availability of computer code

Data collection	No software was used for data collection.
Data analysis	FASTQ files were processed for identifying somatic mutations and copy number alterations using a standardized pipeline involving SurecallTrimmer 4.0.1, BWA-MEM 0.7.17, GATK 4.0.5.2 with Java version 1.8, LocatIt 4.0.1, MuTect 2, CLONET 2, and Nexus Copy Number 9.

For manuscripts utilizing custom algorithms or software that are central to the research but not yet described in published literature, software must be made available to editors/reviewers. We strongly encourage code deposition in a community repository (e.g. GitHub). See the Nature Research guidelines for submitting code & software for further information.

Data

Policy information about availability of data

All manuscripts must include a data availability statement. This statement should provide the following information, where applicable:

- Accession codes, unique identifiers, or web links for publicly available datasets
- A list of figures that have associated raw data
- A description of any restrictions on data availability

Raw sequence data from this study is pending deposit into the NCBI Database of Genotypes and Phenotypes (dbGaP). https://www.ncbi.nlm.nih.gov/projects/gap/cgi-bin/study.cgi?study_id=phs001938.v1.p1. A source data file containing somatic alteration data is provided. MRI and histology files are available upon request to the corresponding author.

Field-specific reporting

Please select the one below that is the best fit for your research. If you are not sure, read the appropriate sections before making your selection.

☒ Life sciences ☐ Behavioural & social sciences ☐ Ecological, evolutionary & environmental sciences

For a reference copy of the document with all sections, see nature.com/documents/nr-reporting-summary-flat.pdf

Life sciences study design

All studies must disclose on these points even when the disclosure is negative.

Sample size	Sample is a single patient from a Phase 2 clinical trial with a total enrollment of 39 patients. The study of intraperson heterogeneity was not a powered study.
Data exclusions	No data were excluded.
Replication	mpMRI was only performed once per time point per patient. Replication of validated clinical imaging procedures is not usually performed. Biopsy and prostatectomy tissue was immunostained at least twice per block at multiple levels to confirm findings. Laser capture microdissected tissue was sequenced only once due to the small volumes of biopsy tissue and residual tumor available for genomic analyses.
Randomization	Randomization was not relevant to this study. Clinical study was single-arm.
Blinding	Blinding was not relevant to this study.

Reporting for specific materials, systems and methods

We require information from authors about some types of materials, experimental systems and methods used in many studies. Here, indicate whether each material, system or method listed is relevant to your study. If you are not sure if a list item applies to your research, read the appropriate section before selecting a response.

Materials & experimental systems

n/a	Involved in the study	
☐	☒	Antibodies
☒	☐	Eukaryotic cell lines
☒	☐	Palaeontology
☒	☐	Animals and other organisms
☐	☒	Human research participants
☐	☒	Clinical data

Methods

n/a	Involved in the study	
☒	☐	ChIP-seq
☒	☐	Flow cytometry
☒	☐	MRI-based neuroimaging

Antibodies

Antibodies used	anti-AR: Cell Signaling, catalog # 5153S; anti-ERG: Abcam, catalog # ab92513; anti-PTEN, Cell Signaling catalog # 9188L; PIN-4 cocktail: Biocare, catalog # PPM225DS
Validation	anti-AR: validated by nuclear staining of luminal cells in normal prostate tissue<br>anti-PTEN: validated by whole-cell staining of normal glands and prior validation in-house using genomically-defined cases with PTEN loss<br>anti-ERG: validated by nuclear staining of blood vessels in prostate tissue and prior validation using genomically-defined cases with TMPRSS2:ER fusion<br>PIN-4 cocktail: validated basal cell staining of normal prostate glands

Human research participants

Policy information about studies involving human research participants

Population characteristics	Eligibility for this clinical trial required a diagnosis of intermediate or high-risk prostate cancer and testosterone levels greater than or equal to 100 ng/dL.
Recruitment	The patient was recruited to this study during his referral to the National Institutes of Health multidisciplinary prostate cancer clinic for a second opinion.
Ethics oversight	National Institutes of Health IRB

Note that full information on the approval of the study protocol must also be provided in the manuscript.

Clinical data

Policy information about clinical studies

All manuscripts should comply with the ICMJEguidelines for publication of clinical research and a completedCONSORT checklist must be included with all submissions.

Clinical trial registration	NCT02430480
Study protocol	Final clinical trial results are not yet published. A trial summary is available here: https://www.cancer.gov/about-cancer/treatment/clinical-trials/search/v?id=NCT02430480. Interim results were conveyed via poster/abstract (DOI: 10.1200/JCO.2019.37.7_suppl.63). A copy of the protocol can be obtained from William L. Dahut, M.D. dahutw@mail.nih.gov
Data collection	mpMRI was performed at study enrollment and prior to surgery. Biopsy was performed following baseline mpMRI. RP tissue was acquired at the conclusion of medical treatment via surgery (robotic-assisted laparoscopic prostatectomy). Tumor volumes were measured by mpMRI and from radical prostatectomy specimens. Sequence data was obtained from biopsy and prostatectomy tissue.
Outcomes	The primary outcome measure was the correlation association between mpMRI-measured volumes of residual tumor and the residual cancer burden measured in the surgical specimen.
